# A case of anisakiasis in the sigmoid colon

**DOI:** 10.1002/ccr3.5445

**Published:** 2022-02-10

**Authors:** Hajime Nakamura, Kunihiro Takanashi, Rie Morita, Akira Sakurada, Yuya Hirata, Yuya Komatsu, Shinichi Katsuki

**Affiliations:** ^1^ Center of Gastroenterology Otaru Ekisaikai Hospital Otaru Japan

**Keywords:** anisakiasis, endoscopy, parasitology, seafood

## Abstract

Colonic anisakiasis is rare because most cases of anisakiasis occur in the stomach. An accurate diagnosis is sometimes difficult because of the rarity and symptom nonspecificity. We should consider the possibility of colonic anisakiasis when examining patients who have a history of consuming raw fish.

## Graphical abstract

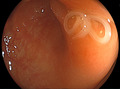

## 1 INTRODUCTION

**FIGURE 1:**
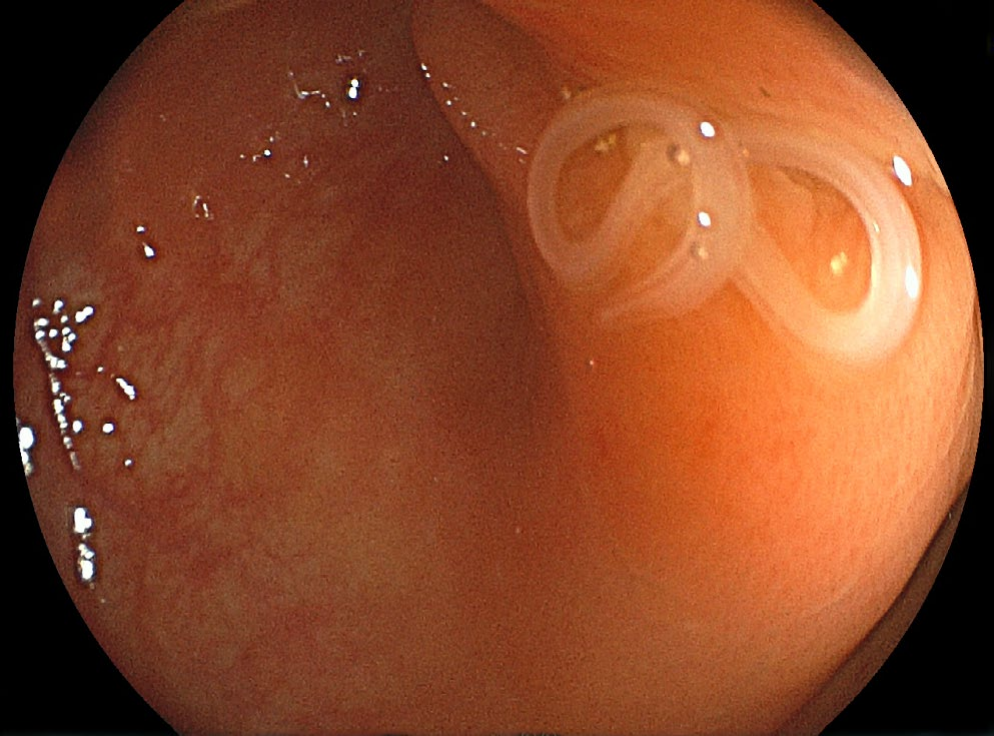
Colonoscopy reveals a grayish‐white worm penetrating the mucosa of the sigmoid colon

**FIGURE 2:**
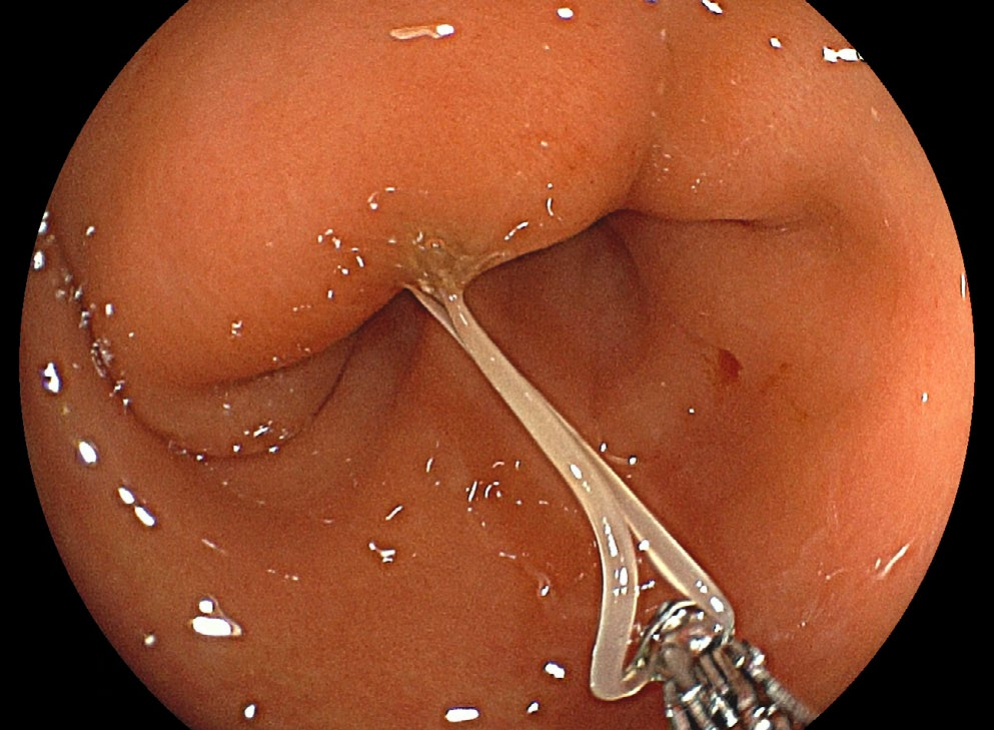
Through the neighboring mucosa was slightly edematous, the larva was removed using biopsy forceps

**FIGURE 3:**
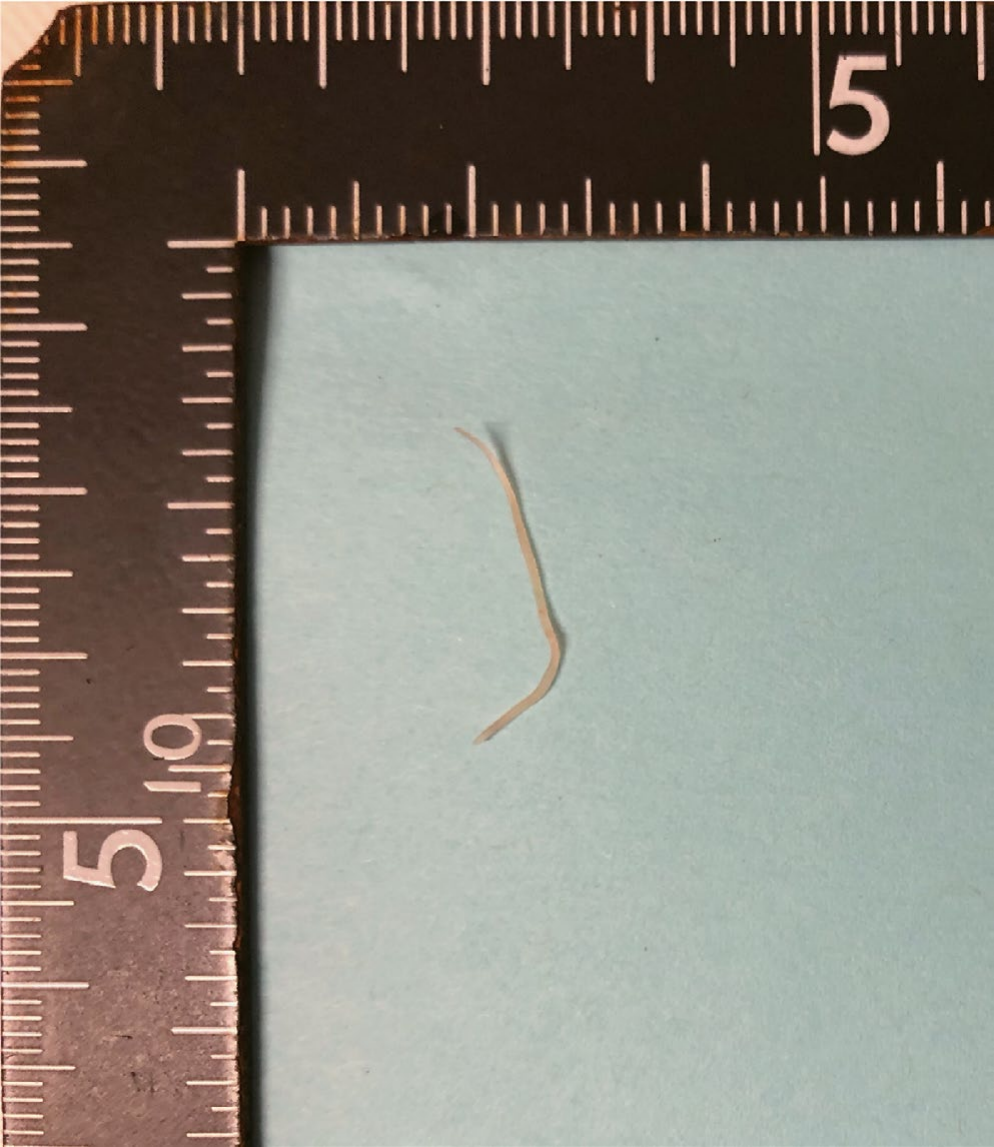
Parasitological evaluation reveals the presence of *Anisakis simple*x with a diameter of 20 mm

A 63‐year‐old man presented to our hospital for further examination of slight abdominal discomfort and a positive fecal occult blood test at his annual health check‐up. We proceeded to perform a colonoscopy, which revealed an approximately 20‐mm grayish‐white worm penetrating the mucosa of the sigmoid colon (Figure 1). The larva was removed using biopsy forceps, and the parasitological evaluation revealed that it was *Anisakis simplex* (Figures 2 and 3). We were informed that the patient had consumed slices of raw squid seven days prior to the examination. Anisakiasis is a human parasitic infection of the gastrointestinal tract caused by the consumption of raw seafood containing *Anisakis simplex*. In Japan, gastric anisakiasis is relatively common because of the tradition of consuming raw fish. Furthermore, the number of anisakiasis cases may reportedly increase throughout the world as the Japanese style of eating raw fish becomes more popular.^1^ Among them, colonic anisakiasis is rare and most of them have been reported in the right side of the colon.^2^ Although the present case is extremely rare since the parasite was found in the sigmoid colon, we should consider the possibility of colonic anisakiasis in patients who have a history of consuming raw fish.

## CONFLICT OF INTEREST

The authors state that they have no conflict of interest.

## AUTHOR CONTRIBUTION

HN and KT: drafted the manuscript. RM, AS, YH, and YK: were involved in the patient's care. SK: supervised the study.

## ETHICAL APPROVAL

This study does not require any ethical committee approval.

## CONSENT

Written informed consent was obtained from the patient to publish this report in accordance with the journal's patient consent policy.

## Data Availability

Data sharing is not applicable to this article as no datasets were generated or analyzed during the current study.
